# A Systematic Review of the Role of Runt-Related Transcription Factor 1 (RUNX1) in the Pathogenesis of Hematological Malignancies in Patients With Inherited Bone Marrow Failure Syndromes

**DOI:** 10.7759/cureus.25372

**Published:** 2022-05-26

**Authors:** Janan Illango, Archana Sreekantan Nair, Rajvi Gor, Ransirini Wijeratne Fernando, Mushrin Malik, Nabeel A Siddiqui, Pousette Hamid

**Affiliations:** 1 Research, California Institute of Behavioral Neurosciences & Psychology, Fairfield, USA; 2 Internal Medicine, California Institute of Behavioral Neurosciences & Psychology, Fairfield, USA; 3 Neurology, California Institute of Behavioral Neurosciences & Psychology, Fairfield, USA

**Keywords:** mutations and polymorphisms, runx1 gene, pathogenesis, hematological malignancies, inherited bone marrow failure syndromes

## Abstract

Somatic *runt-related transcription factor 1* (*RUNX1*) mutations are the most common mutations in various hematological malignancies, such as myelodysplastic syndrome (MDS) and acute myeloid leukemia (AML). Mono-allelic *RUNX1* mutations in germline cells may cause familial platelet disorder (FPD), an inherited bone marrow failure syndrome (IBMFS) associated with an increased lifetime risk of AML. It is suspected that additional *RUNX1* mutations may play a role in the pathogenesis of hematological malignancies in IBMFS. This review aims to study the role of *RUNX1* mutations in the pathogenesis of hematological malignancies in patients with IBMFS. A PubMed database search was conducted using the following medical subject heading (MeSH) terms: “inherited bone marrow failure syndromes,” “hematological neoplasms,” “gene expression regulation, leukemic,” “RUNX1 protein, human,” “RUNX1 protein, mouse,” and “Neutropenia, Severe Congenital, Autosomal recessive.” Three studies published in 2020 were identified as meeting our inclusion and exclusion criteria. Leukemic progression in severe congenital neutropenia was used as a disease model to evaluate the clinical, molecular, and mechanistic basis of *RUNX1* mutations identified in hematological malignancies. Studies in mice and genetically reprogrammed or induced pluripotent stem cells (iPSCs) have shown that isolated *RUNX1* mutations are weakly leukemogenic and only initiate hyperproduction of immature hematopoietic cells when in combination with *granulocyte colony-stimulating factor 3 receptor* (*GCSF3R*) mutations. Despite this, whole-exome sequencing (WES) performed on leukemogenic transformed cells revealed that all AML cells had an additional mutation in the *CXXC finger protein 4* (*CXXC4*) gene that caused hyperproduction of the ten-eleven translocation (TET2) protein. This protein causes inflammation in cells with *RUNX1* mutations. This process is thought to be critical for clonal myeloid malignant transformation (CMMT) of leukemogenic cells. In conclusion, the combinations of *GCSF3R* and *RUNX1 *mutations have a prominent effect on myeloid differentiation resulting in the hyperproduction of myeloblasts. In other studies, it has been noted that the mutations in *GCSF3R *and *RUNX1 *genes are not sufficient for the full transformation of leukemogenic cells to AML, and an additional clonal mutation in the *CXXC4 *gene is essential for full transformation to occur. These data have implicitly demonstrated that *RUNX1 *mutations are critical in the pathogenesis of various hematological malignancies, and further investigations into the role of *RUNX1* are paramount for the development of new cancer treatments.

## Introduction and background

The *runt-related transcription factor 1* (*RUNX1*) gene is known as a critical regulator of em­bryogenesis and definitive hematopoiesis in vertebrates, playing a vital role in the generation of hematopoietic stem cells (HSCs) and their differentiation into the myeloid and lymphoid lineage. The discovery of *RUNX1 *mutations as the cause of familial platelet disorder (FPD) was pivotal to understanding the implications of this gene in hematological malignancies. FPD is an inherited bone marrow failure syndrome (IBMFS) with quantitative and qualitative platelet abnormalities and a high predisposition to acute myeloid leukemia (AML) [1,2]. IBMFS are genetic disorders characterized by cytopenia and hypoproliferation of one or more cell lineages in the bone marrow [1]. The production of blood cells (erythrocytes, granulocytes, and platelets) is compromised because of the mono-allelic gene mutation in one of certain bone marrow genes. Besides FPD, the other most common IBMFSs include Fanconi anemia (FA), Diamond-Blackfan anemia (DBA), Shwachman-Diamond syndrome (SDS), and severe congenital neutropenia (SCN) [3]. Patients with IBMFSs show a predisposition to developing hematological complications, such as myelodysplastic syndrome (MDS) or AML [3]. MDS is a pre-leukemic state defined by the presence of refractory cytopenia or refractory cytopenia with an excess of blasts (5-29%) in the bone marrow. AML is a blood cancer that is characterized by rapid leukemic blast cell growth and the presence of more than 30% myeloid blasts in the bone marrow [2].

Recent studies have shown that *RUNX1 *germline mutations in patients with IBMFS are like ac­quired or somatic *RUNX1* mutations that were found in myeloid malignancies, particularly in MDS and AML [3]. It has become clear that somatic *RUNX1 *mutations are more prevalent in MDS/AML that is secondary to IBMFS, such as FA and SCN. Unlike acquired MDS/AML, these forms of secondary MDS/AML are often refractory to treatment, resulting in a poor prognosis. Because the somatic mutation of *RUNX1 *was first identified in MDS and AML, *RUNX1 *has become known to be one of the most frequently mutated genes in a variety of hematological malignancies [4].

Despite recent research having demonstrated the strong association of *RUNX1 *mutations in a variety of hematological malignancies, it is unclear how *RUNX1 *mutations contribute to the pathogenesis of hematological malignancies in IBMFS. What are the fre­quencies of different *RUNX1 *mutations in various subgroups of hematological malignancies, as well as their impact on progno­sis? Furthermore, is there any potential for the development of new cancer therapies following recent findings regarding the role of *RUNX1 *in the malignant transformation [5]?

In this article, we summarize new research on the role of *RUNX1 *mutations, published in February 2020 by three different groups [6-8]. They performed different experiments in human, mouse, and induced pluripotent stem cell (iPSC) models to decipher the role of the *RUNX1 *gene in the malignant transformation of IBMFS; the mechanisms of pathogenesis; clinical and molecular characteristics of *RUNX1 *mutations; and the potential for the treatment of cancers. The mouse and iPSC models suggested that secondary *RUNX1 *mutations in clones with granulocyte colony-stimulating factor 3 receptor (GCSF3R) mutations are weakly leukemogenic and that an additional clonal mutation in the *CXXC finger protein 4* (*CXXC4*) gene is required for the full transformation to AML [9]. Mutations in the *CXXC4 *gene lead to the hyperproduction of inflammatory proteins called the ten-eleven translocation (TET2) proteins. This inflammation, in combination with the *RUNX1 *mutations, drives the development of myeloid malignancies [10]. The other pathogenic mechanisms wherein *RUNX1 *mutations may initiate tumor cell proliferation 18 are the inhibition of the p53 pathway and hypermethylation of the promoter of *Wingless and Int1* (*WNT*) inhibitor gene called *secreted frizzled-related protein 2* (*SFRP2*) [11,12].

These discoveries may have the potential to aid the development of new therapeutic strategies. Specifically, immunotherapy may be employed for suppression of the excessive immune response to hyperproduction of TET2 proteins. The other potential therapeutics, such as mouse double minute 2 (MDM2) and poly adenosine diphosphate-ribose polymerase (PARP) inhibitors, may be used to inhibit the hyperactivation of the p53 pathway or hypersensitivity to DNA damage resulting from *RUNX1 *mutations [11]. Because the presence of *RUNX1 *mutation represents a poor prognostic factor in patients with MDS or AML, the investigation of various biomarkers is critical as they may detect the clones with *RUNX1 *mutation, in the early stages of leukemic progression [7].

## Review

Methodology

Search Strategy

The PubMed online database search was used to select the articles which are included in this review. The findings were reported according to Preferred Reporting Items for Systematic Reviews and Meta-Analyses (PRISMA) guidelines. The following medical subject heading (MeSH) parameters were used: “inherited” and “bone marrow” and “failure” and “syndromes.” This search resulted in 5,051 articles.

Selection Criteria

**Figure 1:**
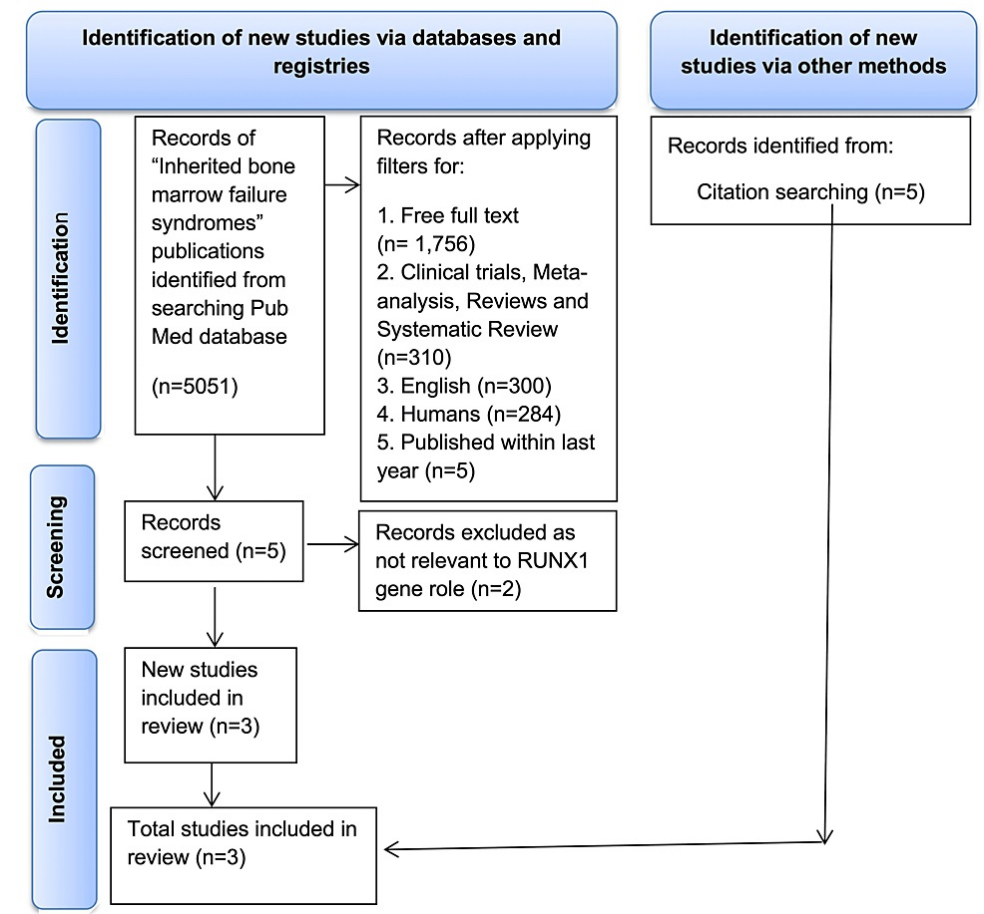
Preferred Reporting Items for Systematic Reviews and Meta-Analyses (PRISMA) diagram. RUNX1: runt-related transcription factor 1

The identified articles were further filtered. The review selected only articles that met the following criteria: (1) papers published between January and December 2020; (2) free full-text available; (3) papers written in English; and (4) studies conducted on human participants. Among screened articles, only clinical trials, meta-analyses, randomized controlled trials, and systematic reviews were included. Five citations from other sources were not included because they were not relevant to the topic. To further select the articles, we included the following MeSH terms: “hematologic neoplasms,” “gene expression regulation, leukemic,” “RUNX1 protein, human,” and “Neutropenia, Severe Congenital, Autosomal recessive.” Any articles that were not relevant to the role of the *RUNX1* gene were excluded. These criteria allow comparison between articles; however, it should be noted that differing lab protocols between studies prevents validation of results using the same assessment tool. A systematic search review is reported using the PRISMA 2020 guidelines [13]. The diagram is presented in Figure 1.

Results

The selected articles were used to evaluate the clinical and molecular characteristics of *RUNX1 *mutation in various types of hematological malignancies, the mechanisms of pathogenesis caused by *RUNX1 *mutations, and potential therapeutic strategies for hematological malignancies with *RUNX1 *mutations.

Clinical and Molecular Characteristics of RUNX1 Mutation in Hematological Malignancies

*RUNX1 *gene has multiple biological functions in the human body. It regulates hematopoiesis, the cell cycle and genome stability, the p53 signaling pathway, apoptosis, and ribosomal biogenesis. During hematopoiesis, this gene controls the development of HSCs and their differentiation in different lineages. The transition from the G1-S to the G2/M phase of the cell cycle is facilitated by *RUNX1*. This gene controls cellular proliferation and differentiation via direct regulation of transcription, achieved by binding promoters of the genes that are encoding ribosomal RNA/proteins. According to recently published data, somatic mutations of *RUNX1 *were observed in various types of hematological malignancies. We present the frequency of *RUNX1 *mutations in various types of hematological malignancies in Table 1 below.

**Table 1:** The frequency of RUNX1 mutations in various types of hematological malignancies. FPD: familial platelet disorder; AML: acute myeloid leukemia; MDS: myelodysplastic syndrome; CMML: chronic myelomonocytic leukemia; MPN: myeloproliferative neoplasm; ALL: acute lymphoblastic leukemia; CBMF: congenital bone marrow failure; FA: Fanconi anemia; SCN: severe congenital neutropenia

References	Hematological malignancies	Subtypes	Frequency of *RUNX1* mutations (%)
Latger-Cannard et al. [14]	FPD/AML		>70 families
Sood et al. [5]	FPD/AML		>70 families
Vormittag-Nocito et al. [15]	FPD/AML		>70 families
Gaidzik et al. [16]	AML	Primary AML	5.6–17.9
Cazzola et al. [17]	MDS		10
Haferlach et al. [18]	MDS		10
Steensma et al. [19]	MDS		10
Kuo et al. [20]	CMML		32.1–37
Tsai et al. [21]	CMML		32.1–37
Grossmann et al. [22]	ALL	T-ALL	15.5–18.3
Zhang et al. [23]	ALL	ETP-ALL	15.6
Singhal et al. [24]	Radiation t-MDS/AML		15.7–39
Cerquozzi et al. [25]	MPN	Ph^-^ MPN	10.3–37.5
Branford et al. [26]	MPN	Ph^-^ MPN	12.9–33.3
Baer et al. [27]	MPN	MPN-Eo	32–71
Strati et al. [28]	MPN	MPN-Eo	32–71
Chao et al. [29]	CBMF	FA	20.7–31.3
Quentin et al. [30]	CBMF	FA	20.7–31.3
Skokowa et al. [31]	CBMF	SCN	64.5

Most frequently, somatic mutations of *RUNX1 *were associated with the development of myeloproliferative neoplasm (MPN) (10.3-37.5%) and chronic myelomonocytic leukemia (CMML) (32.1-37%). Despite this, the association between *RUNX1 *somatic mutations and MDS was only 10%.

The Mechanisms of Pathogenesis Caused by RUNX1 Mutations

**Figure 2:**
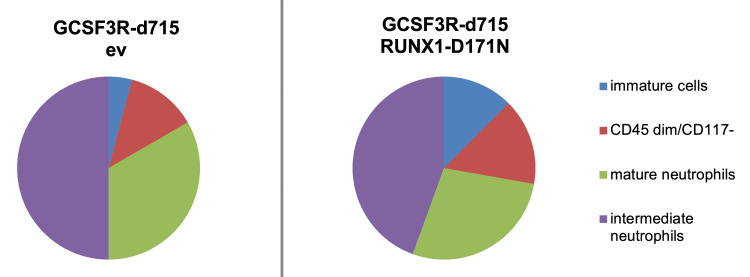
Myeloid differentiation of GCSF3R-d715/RUNX1-D171N cells compared to GCSF3R-d715/ev cells without RUNX1-D171N lentiviral expression vector or with an ev. GCSF3R: granulocyte colony-stimulating factor receptor; RUNX1: runt-related transcription factor 1; ev: empty vector

In the selected studies, the different mechanisms of pathogenesis caused by *RUNX1 *mutations were characterized. It has been shown that loss of *RUNX1 *function causes inhibition of differentiation of HSCs. Therefore, in pre-leukemia, we found expansion of HSCs and progenitor cells. *RUNX1 *muta­tions may attenuate the G1-S phase and enhance the proliferation of hematopoietic cells that occur during the mitotic phase of the cell cycle (G2/M) [7]. The mutations can also result in genomic instability, leading to increased DNA damage and impaired DNA repair. Some mutations in *RUNX1 *are associated with alterations of signaling pathways, such as WNT and p53. Hypermethylation of the WNT inhibitor gene promoter, *SFRP2*, can lead to aberrant activation of the WNT signaling pathway and leukemogenesis in AML. When functioning normally, the *RUNX1 *gene acts to increase transcrip­tional activity of the p53 signaling pathway, in response to DNA damage caused by exposure to different agents such as chemicals, radiation, and toxins. Mutations in *RUNX1 *may lead to defects in p53-mediated apoptosis/DNA repair/cell cycle regulation resulting in tumorigenesis. Furthermore, loss-of-function mutations of *RUNX1 *may aid tumor-initiating cells in hematological malignancies via inhibition of p53 signaling and apoptosis, among other mechanisms. Such mutations have reduced ribosomal biogenesis in HSCs and directed to malignant proliferative processes in the pre-leukemic stage [6]. In vivo studies, administration of amino acid L-leucine to patients with DBA resulted in loss-of-function mutations in ribosomal protein genes. Research into iPSC confirmed that the introduction of the mutated *RUNX1 *gene into CD34+CD45+ cells via lentivirus can stimulate receptor which binds the granulocyte colony-stimulating factor 3 receptor (GCSF3R) and initiates the production of immature cells. The percentage of immature cells was significantly increased when compared to the percentage in empty vector (ev) control studies. The myeloid differentiation of GCSF3R-d715/RUNX1-D171N and GCSF3R-d715/ev cells without RUNX1-D171N lentiviral expression vector or with an ev is presented in Figure 2.

Potential Therapeutic Strategies for RUNX1-Mutated Cases of Hematological Malignancies

**Figure 3:**
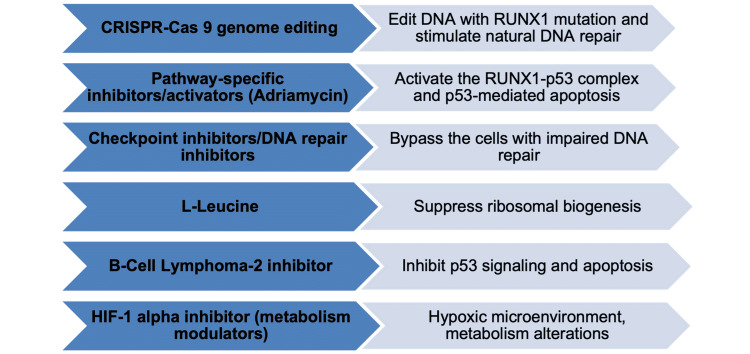
Potential therapeutic strategies in hematological malignancies with RUNX1 mutations. CRISPR-Cas9: clustered regulatory interspaced short palindromic repeats-associated genes; HIF-1 alpha: hypoxia-inducible factor 1-alpha; RUNX1: runt-related transcription factor 1

Clinical trials demonstrated potential therapeutic strategies for *RUNX1* mutated hematologic malignancies. Based on the current *RUNX1 *roles in human hematopoiesis, various therapeutic options were developed. Thus far, the different DNA repair inhibitors can be useful in the M phase of cell cycle repair or bypassing the cells with damage because *RUNX1 *mutations lead to DNA damage and impaired DNA repair [32]. In addition, adriamycin as an antineoplastic drug can stimulate the *RUNX1*-p53 complex which is important in the activation of p53-mediated apoptosis [11]. L-leucine can be used to improve anemia in the genetic DBA mouse models and DBA patients. This agent is a potent stimulator of protein translation that is initialized by the activation of the mammalian target of rapamycin (mTOR) protein kinase. This kinase stimulates protein synthesis [33]. Another agent, clustered regulatory interspaced short palindromic repeats-associated genes (CRISPR-Cas) can be used as a genomic targeted treatment as this agent can edit the *RUNX1* gene by cutting pieces of DNA where *RUNX1 *mutations are, followed by stimulating natural DNA repair [6]. Finally, hypoxia-inducible factor 1α (HIF-1α) in­hibitor can potentially treat various hematological malignancies as a modulator of cell metabolism. MDS and other hematological malignancies are in hypoxia-like status and produce their energy through the tricarboxylic acid (TCA) cycle. The use of HIF-1α in­hibitor can suppress the TCA cycle and modulate it into an aerobic metabolic pathway called glycolysis through which the normal cells are supplied with energy. The recent studies proposed therapeutic strategies that employed the different pathophysiological mechanisms to correct the *RUNX1 *mutations, as shown in Figure 3.

Discussion

The *RUNX1 *gene plays essential roles in a wide range of biological processes, including the development of HSCs, cell proliferation, megakaryocyte maturation, T lymphocyte-lineage differentiation, and apoptosis. It is not surprising that *RUNX1 *dysfunction is associated with the development of IBMFSs and various hematological malignancies [7,21,34].

Previous studies have shown that *RUNX1 *is one of the most frequently mutated genes in hematological malignancies. *RUNX1 *mutations account for about 10-15% of all somatic mutations that have been detected in MDS [21,35]. The incidence of *RUNX1 *mutations in CMML and chronic myelogenous leukemia (CML) is even higher, ranging from 32.1% to 37%, respectively [36]. *RUNX1 *mutations have also been reported in 14% of patients with MPN, 15.6% of patients with acute lymphoblastic leukemia (ALL), and 10.3-37.5% of AML patients. Importantly, these studies have shown that mutated *RUNX1 *can be used as an independent prognostic factor for event-free survival (EFS), relapse-free survival (RFS), or overall survival (OS) in hematological malignancies [37]. Therefore, AML patients with *RUNX1 *mutations had worse prognosis, resistance to chemotherapy, and inferior EFS, RFS, and OS. Reduced OS was also observed in high-risk MDS patients with *RUNX1 *mutations who had poor clinical outcomes and shorter latency for progression to secondary AML [38,39].

Little is known about the role of the *RUNX1 *gene in the development of secondary somatic mutations in patients with IBMFSs and how these mutations lead to hematological malignancies. The data have shown that individuals with IBMFSs, such as FPD and FA, have a high lifetime risk (30-44%) of developing MDS and AML [29,30]. Among FA-associated MDS or MDS/AML patients, *RUNX1 *mutations were detected in the range from 20.7% to 31.25%, respectively. In SCN-MDS/AML patients *RUNX1 *mutations were seen at the highest rate of up to 64.5% which revealed that these types of mutations are the most frequent somatic secondary mutations in SCN-MDS/AML [31,40,41]. Given that the patients with SCN are more prone to develop somatic *RUNX1 *mutations, SCN/AML has been recognized as an important model to further investigate the role of secondary *RUNX1 *mutations in the molecular pathogenesis of hematological malignancies. SCN is an IBMFS classified by severe neutropenia and life-threatening infections such as fungal infections or bacterial sepsis [40]. The most frequent mutated gene is* encoding neutrophil elastase* (*ELANE*). The treatment consists of life-long administration of GCSF3 that successfully alleviates the neutrophil counts [42]. As is common with other forms of IBMFSs, SCN patients have a high risk of developing MDS or AML. The incidence of developing MDS or AML directly correlates to the number of years on GCSF3. Therefore, after 15 years on GCSF3, the incidence of developing MDS or AML is 21% [31]. The majority of SCN patients with leukemic progression develop hematopoietic clones with somatic mutations in *GCSF3R*, resulting in a truncated form of *GCSF3R *[42]. It is important to note that these clones can persist for several months or years before MDS or AML becomes symptomatic, raising the question of how these *GCSF3R *mutants contribute to the malignant transformation of SCN [31,41]. Given this, a mouse model was used to study the role of *RUNX1*. In this study, a truncated *GCSF3R *(GCSF3R-D715) identical to the mutant *GCSF3R *form in SCN patients was expressed in mice [43]. In addition, a lentiviral expression vector was used to express *RUNX1*-mutant D171N in conjunction with an enhanced green fluorescent protein (eGFP) [8]. The mouse bone marrow (BM) cells with expressed *GCSF3R-D715* mutation were subsequently serially transplanted into wild-type recipients. Before transplantation, the recipients were treated either three times per week with GCSF3 or with peripheral blood solvent (PBS) control. Primary recipients who were treated with GCSF3 and transplanted with *GCSFR3-RUNX1*-mutant BM cells developed myeloblasts in peripheral blood (PB) that were sustained for at least 30 weeks. None of these mice developed symptoms of AML, suggesting that the elevated myeloblasts in the PB reflected a pre-leukemic state rather than a fully transformed state. However, upon transplantation in secondary and tertiary recipients, mice developed *GCSF3R-RUNX1*-mutant AML. Whole-exome sequencing (WES) was performed on lin-c-kit (LK) cells and revealed that AML cells from the secondary and tertiary recipients had seven-fold higher expressions of *CXXC4* mutations than the cells from the primary recipient. Recently, *CXXC4 *mutations have also been detected in human AML cases [9]. It seems that *CXXC4 *mutations enhance the production of TET2 protein which is known to be an inflammatory factor and has a similar role to interferon-gamma, interleukin-6, and others. Interferon-gamma and interleukin-6 are cytokines that are produced in response to infections and tissue damage, with pro- and anti-inflammatory effects. Hyperproduction of TET2 leads to inflammatory processes that may play an important role in the development of myeloid malignancy involving *RUNX1 *mutations [10]. In conclusion, isolated *RUNX-Runt homology domain* (*RHD*) mutations are only weakly leukemogenic and an additional clonal mutation that reduces levels of TET2 is what drives the full transformation to AML [8,32]. The data suggest the need for further investigation into the somatic *RUNX1 *mutations in HSPCs that already harbour a *GCSF3R *nonsense mutation. To achieve this, a CRISPR/Cas9-based strategy was used to introduce a patient-derived *GCSF3R *nonsense mutation into iPSC. CRISPR-Cas9 is a technology used for removing, adding, or altering sections of the DNA. After culturing iPSC, CD34+CD45+ cells were transduced using a lentivirus to express the *RUNX1-RHD D171N* mutant. The experiments confirm that the combinations of *GCSF3R* and *RUNX1 *mutations have a moderate effect on myeloid differentiation and result in an increasing number of myeloblasts. These findings corroborate the findings in the mouse model and suggest that secondary *RUNX1 *mutations in clones with *GCSF3R *mutations are not sufficient to fully transform to AML.

Most of the *RUNX1 *mutations are mono-allelic, such as in FPD, an IBMFS resulting in a predisposition to leukemia [1,2]. Germline *RUNX1 *mutations are dominant-negative mutations and correlate to a higher risk of developing hematological malignancies compared to RUNX1 loss-of-function mutations [5-8]. It is important to note, however, that such germline mutations alone are not sufficient for the development of leukemia and additional mutations in *RUNX1 *(bi-allelic mutations) or epigenetic modifiers, splicing factors, or tumor suppressors are required to induce myeloid malignancies [1,4].

It has been observed that mutations in *RUNX1 *are associated with alterations of p53 and other signaling pathways, such as WNT, bone morphogenetic proteins (BMP), transforming growth factor-beta (TGF-β), rat sarcoma-the extracellular signal-regulated kinase (RAS-ERK), Hippo-yes-1-associated protein (YAP1), and Notch. Unlike mono-allelic mutations, loss-of-function mutations of *RUNX1 *are responsible for initiating tumor cell proliferation by inhibiting the p53 signaling pathway and apoptosis. The p53 pathway is activated in DNA damage and is responsible for DNA repair. *RUNX1 *increases the transcriptional activity of p53, potentially via up-regulation of p300-mediated acetylation of p53. *RUNX1 *mutations lead to a reduction of p53-mediated apoptosis [11]. The WNT pathway is important for cellular proliferation and differentiation, with aberrant activation of this pathway being reported in various tumors. *RUNX1* mutations were closely associated with hypermethylation of the promoter of one of the WNT inhibitor genes (*SFRP2*) in AML. It was suggested that the WNT inhibitor hypermethylation might lead to aberrant activation of the WNT signaling pathway. It is suggested that mutation in the *RUNX1 *gene can interact with the *SFRP2 *gene which is known as an inhibitor gene responsible for the suppression of the WNT signaling pathway. Due to interaction with genetic alterations, the hypermethylation of *SFRP2 *gene promoter is initiated and leads to leukemogenesis where cellular proliferation and differentiation are uncontrolled [12].

Strengths and limitations

This review has highlighted the importance of studying the role of somatic *RUNX1 *mutations in the pathogenesis of hematological malignancies and the potential implications in the development of oncological therapies. This review does, however, had some limitations. First, the results presented in this review were collected from only three articles that were published over the limited time frame of one year. In addition, we included only articles that were available in the PubMed database and in both free text format and English language. This review did not apply the same assessment tools such as the lab protocols for conducting experiments. Variations between lab protocols did not allow the comparison of study results. In all the articles included, the scope of the study was the role of *RUNX1 *mutations in animal and human disease models, including only SCN and FA as the IBMFS representatives without knowing if *RUNX1* mutations may contribute to the development of malignancies in other IBMFS. A broader literature search and greater inclusion of studies about *RUNX1 *mutations in pathogenesis in other IBMFS may better represent and validate the inferences from this review.

## Conclusions

*RUNX1* plays important role in responding to cellular stress, maintaining genomic stability, and ensuring cellular quality control. Dysregulation of *RUNX1 *expression contributes to the pathophysiology of IBMFS and cancer predisposition. This review revealed important clinical implications of *RUNX1*. Mutations in the *GCSF3R *factor are associated with granulocyte colony-stimulating factor treatment and may lead to cancer predisposition in patients with SCN. Combinations of *GCSF3R *and *RUNX1 *mutations can activate the p53 signaling pathway and lead to the accumulation of immature cells. Studies in mice have shown that *RUNX1 *and *GCSF3R *mutations found in combination do not lead to leukemic progression without additional inflammation. These discoveries may be utilized in the development of new therapeutic strategies. The use of immunotherapy or different inhibitors (MDM2, PARP) has shown promise in preventing p53 pathway activation and hypersensitivity to DNA damage of cells containing somatic *RUNX1 *mutations. Further research may lead to the discovery of biomarkers for early detection of leukemic progression, promoting a deeper understanding of molecular mechanisms by which *RUNX1 *mutations contribute to hematological malignancies and the development of new therapeutic interventions.
